# The impact of excess weight and body fat on clinical outcomes of immune checkpoint inhibitors according to gender

**DOI:** 10.1007/s40618-025-02722-1

**Published:** 2025-10-27

**Authors:** Marta García-Goñi, María Olmedo, Adriana García-Goñi, Francisco Guillén-Grima, Juan C. Galofré, Miguel Fernández de Sanmamed

**Affiliations:** 1 Present Address: Department of Endocrinology and Nutrition, Clínica Universidad de Navarra, Pamplona, Spain; 2 Department of Oncology, Clínica Universidad de Navarra, Pio XII, 36, Pamplona, 31008 Spain; 3 Departament of Oncology, Hospital Universitario Ramón y Cajal, Madrid, Spain; 4 Department of Preventive Medicine, Clínica Universidad de Navarra, Pamplona, Spain; 5 IdiSNA (Instituto de investigación Sanitaria de Navarra), Pamplona, Spain

**Keywords:** Excess weight, Excess body fat, Gender, Survival, Immune-related adverse events, Anti-PD-(L)1

## Abstract

**Background:**

The impact of body-mass index (BMI) on immune checkpoint inhibitor efficacy and toxicity has not been clearly characterized. We analyzed the association between BMI, and body fat (%BF), with the efficacy and toxicity of ICIs across three solid tumors in a real-life setting.

**Methods:**

Melanoma, lung and urothelial cancer patients treated with ICIs at our institution were included. BMI (kg/m2) and %BF (CUN-BAE) were calculated retrospectively. We studied the association between BMI/%BF and objetive-response-rate (ORR), progression-free survival (PFS), overall survival (OS) and immune-related adverse events (irAEs).

**Results:**

Among the 356 patients included, 177 (49.7%) had a BMI ≥ 25 kg/m2. Mean BMI was 25.3 ± 4.2 kg/m2, and %BF 30.5 ± 6.3%. ORR was achieved in 155 patients (46.8%). Median PFS and OS was 4 and 11 months, respectively. There were no differences in ORR across BMI categories. In contrast, normal %BF was associated with better ORR in men (81.8% vs. 41.7%, *p* = 0.024), but not in women (*p* = 0.074). Additionally, no association was observed between BMI/%BF and irAEs (*p* = 0.762). Notably, those developing any-grade irAEs showed better ORR (*p* < 0.001), PFS (HR 1.6, *p* < 0.001) and OS (HR 1.7, *p* < 0.001), even adjusting by BMI/%BF, age, gender, primary tumor or ICI regimen.

**Conclusions:**

Our results suggest that in patients with advanced cancers treated with ICIs, BMI was not correlated with clinical outcomes or survival. However, men with normal %BF showed better ORR compared to men with excess-%BF, but this pattern was not observed in women. These findings support to consider gender and body composition as stratification factors in trials.

## Introduction

The development of immunotherapy, mainly immune-checkpoint-inhibitors (ICIs), has changed the paradigm in cancer treatment, showing an increase in survival in multiple metastatic tumors [1]. However, a tiny proportion of patients exhibited a sustained response to ICIs, with many of these patients subsequently developing primary or secondary resistance. Furthermore, response rates vary among different malignancies, such as non-small cell lung cancer and renal carcinoma, with a response rate lower than 20% [2, 3] and 30% [4, 5], respectively.

This observation and approach to the application of precision medicine [6] make it essential to better understand the mechanisms of response to ICIs. It is crucial to recognize biomarkers as predictors of individual response to a specific immunotherapy strategy [7]. In this scenario, several studies have been carried out to identify patients who might benefit from these new therapies. Currently, there is a robust line of research focused on tumor microenvironment-dependent parameters [7, 8] such as PD-L1 expression [9], tumor mutational burden (TMB) [10], microsatellite instability (MSI), the burden of tumor-infiltrating lymphocytes (TILs) [11] or resistance mutations (such as STK11 or B-catenin) [12]. However, there is little information on patient-related factors such as gender, body weight assessed by BMI or estimated percetage of body fat (BF).

We aimed to evaluate the impact of patient BMI and %BF on treatment response and survival outcomes of patients with three different selected aggressive malignancies treated with ICIs, combined or in monotherapy. As it has also been suggested that obese patients tend to develop more immune-related adverse events (irAEs), we aimed to analyze if this occurred in our cohort and, if so, if it impacted clinical outcomes.

## Materials and methods

### Study population and data

We conducted a study of 356 patients with three different histologically-confirmed solid tumors (melanoma, lung and urothelial carcinoma) treated with at least one cycle of immunotherapy-based regimens with anti-PD-1 (Nivolumab or Pembrolizumab) or anti-PD-L1 (Atezolizumab) monoclonal antibodies (mAbs) at the Clínica Universidad de Navarra (Pamplona, Spain) between 2012 and 2022.

Patients were classified according to pretreatment BMI into two groups, normal weight (< 25 kg/m^2^) or excess-weight (≥ 25 kg/m^2^, including overweight and obese patients), following the WHO and the National Institute of Health (NIH) criteria [13, 14].

%BF was calculated according to CUN-BAE formula [15, 16]. CUN-BAE (Clínica Universidad de Navarra-Body Adiposity Estimator, Prediction equation) is a validated, easy-to-apply method based on age, sex and BMI for estimating %BF. Ranges of %BF were defined as normal (lean) < 20% in men and < 30% in women and obese ≥ 20% in men and ≥ 30% in women (these cutoff values are widely used in studies involving Caucasian populations to define normal vs. excess body fat) [16]. Patients were also divided according to %BF into two categories (normal vs. excess-%BF) and, in order to better analyze the magnitude of the association, we made quartiles and quintiles according to BMI and %BF.

Response to treatment was determined using the modified Response Evaluation Criteria in Solid Tumors (RECIST v1.1). Patients were categorized according to their best response to ICIs as having complete response (CR), partial response (PR), stable disease (SD) and progressive disease (PD). RECIST could not be assessed in twenty-five patients because they died without imaging tests being performed, so they were excluded for the outcomes analysis (*n* = 331).

A database with coded information on patients was created to ensure confidentiality. All patients signed an informed consent form approved by the Clínica Universidad de Navarra Research Ethics Committee (approval 111/2010).

All patients were followed from the first day of treatment until the last follow-up date or the patient’s death. Baseline laboratory tests were performed according to routine clinical practice. Body CT scans were performed every 8–12 weeks or as clinically indicated.

Baseline and follow-up collected data for each subject included demographic information, primary tumor site, Eastern Cooperative Oncology Group (ECOG) performance status, number of distant metastases, number of prior lines of systemic therapy, number of immunotherapy cycles and whether treatment was given as monotherapy or in combination with chemotherapy, their best overall response (BOR), objetive response rate (ORR), presence or absence of irAEs, and tumor PD-L1 expression. Each patient’s living status (dead or alive), disease progression, date of disease progression, date of death, or last follow-up visit were recorded for survival analyses.

Treatment efficacy was measured using objective response rate (percentage of patients who had a partial or complete response to the treatment; ORR = CR + PR/total), progression-free surivival (PFS), and overall survival (OS). Progression-free survival (PFS) was calculated from the initiation of ICI until the date of disease progression. Overall survival (OS) was calculated from the beginning of ICI until the date of death. Individuals who had not progressed or were still alive at the time of data analysis were censored at the date of last follow-up.

IrAEs were defined according to the criteria published in previous studies [17] and their degree was established following the Common Terminology Criteria for Adverse Events version 5.0 (CTCAE-5.0) [18], considering immunotoxicity any grade.

### Statistical analysis

Descriptive statistics were used to analyze the characteristics of the patients, and univariate analyses were conducted to examine differences between BMI (< 25 kg/m^2^ vs. ≥25 kg/m^2^) and %BF (< 20% vs. >20% in men and < 30% vs. >30% in women). Descriptive data are presented as relative frequencies for qualitative variables. Continuous variables are reported as mean ± standard deviation (SD) or median and interquartile range (IQR) for normal and not normal distributed variables, respectively. Shapiro-Wilk test was performed to assess normality.

ORR was defined as the percentage of patients who present any response to treatment (CR and PR) assessed between the first day of treatment to progression, death, or last follow-up. Chi-square Test and Odds Ratio were performed to determine the association between BMI or %BF categories and ORR.

To determine the relationship between the development of any-grade irAEs and the achievement of their ORR, Pearson Chi-square Test and Odds Ratio were also performed. A Student’s t-test was performed to analyze the association between BMI or %BF with quantitive variables. Mann-Whitney U and Kruskal Wallis H tests were used for non-normally distributed variables.

Kaplan–Meier curves were appraised from treatment initiation to death or last date of follow-up and compared by the log-rank test. Cox models were developed using baseline variables for PFS and OS. Multivariate logistic regression was conducted to identify predictors of ORR, irAEs development, progression, or death.

The statistical significance threshold was set to a two-tailed p-value of 0.05. Statistical analyses were conducted using IBM SPSS Statistics 25.0 software (SPSS Inc.).

## Results

### Patient characteristics

Three hundred fifty-six patients treated with antiPD-(L)1 agents at the Clínica Universidad de Navarra were included. The distribution of baseline characteristics between excess-weight and normal-weight patients are summarized in Table 1. Lung cancer was more prevalent in the BMI < 25 kg/m^2^ group (normal weight), whereas melanoma was more prevalent in the BMI ≥ 25 kg/m^2^ group (excess-weight). The median number of immunotherapy cycles received was 5 (IQR 3–10). Overall, 261 patients (74%) received ICIs as monotherapy, whereas 92 patients (26%) received them in combination with chemotherapy (Table 1).

**Table 1:** Baseline characteristics and differences between excess-weight (BMI ≥ 25 kg/m^2^) and normal BMI (< 25 kg/m^2^)

*N* (%)		*Total* (*n = 356*)	*Excess weight* (*n = 177*)	*Normal weight* (*n = 179*)	*p* value
Age, mean (y)		64.57	65.28	63.87	*P* = 0.205
Gender	Female	105 (29.5)	29 (16,4)	76 (42.5)	*P* < 0.001
	Male	251 (70.5)	148 (83,6)	103 (57.5)	
Percentage of body fat (%BF)	Female Male	36.41 28.04	43.05 31.03	33.88 23.73	*P* < 0.001 *P* < 0.001
Smoking habit	Current	62 (18)	102 (60.3)	32 (18.3)	*P* = 0.789
	Former	209 (60.8)	30 (17.8)	107 (61.1)	
	Never	73 (21.2)	37 (21.9)	36 (20.6)	
Cancer type	Lung	220 (61.8)	108 (61)	112 (62.6)	*P* = 0.471
	Melanoma	74 (20.8)	41 (23.2)	33 (18.4)	
	Urothelium	62 (17.4)	28 (15.8)	34 (19)	
PD-L1 status in NSCLC	≥ 50%	13 (11.1)	8 (15.4)	5 (7.7)	*P* = 0.188
	< 50%	104 (88.9)	44 (84.6)	60 (92.3)	
Treatment	Nivolumab	120 (33.7)	65 (36.7)	55 (30.7)	*P* = 0.445
	Atezolizumab	104 (29.2)	51 (28.8)	53 (29.6)	
	Pembrolizumab	132 (37.1)	61 (34.5)	71 (39.7)	
Treatment dose	Fixed dose	216 (67.9)	108 (69.2)	108 (66.7)	*P* = 0.624
	Per kg	102 (32.1)	48 (30.8)	54 (33.3)	
Previous neoadjuvant or adjuvant treatment	Yes	121 (34.5)	61 (35.1)	60 (33.9)	*P* = 0.584
ICI monotherapy or combination	No Monotherapy Combination	230 (65.5) 261 (77) 92 (26)	113 (64.9) 137 (77.4) 40 (22.6)	111 (66.1) 124 (70.4) 52 (29.6)	*P* = 0.137
ECOG PS	0–1	325 (91.3)	166 (93.8)	159 (88.8)	*P* = 0.097
	≥ 2	31 (8.7)	11 (6.2)	20 (11.2)	
Immune-related Adverse Events	Yes	112 (31.5)	57 (32.8)	55 (31.2)	*P* = 0.764
	No	244 (68.5)	120 (67.8)	124 (69.3)	
Diabetes Mellitus	Yes	50 (14)	31 (17.5)	19 (10.6)	*P* = 0.061
	No	306 (86)	146 (82.5)	160 (89.4)	
Dyslipidemia	Yes	131 (36.8)	73 (41.2)	58 (32.4)	*P* = 0.084
	No	225 (63.2)	104 (58.8)	121 (67.6)	
Hypertension	Yes	143 (40.2)	89 (50.3)	54 (30.2)	*P* < 0.001
C-Reactive Protein, mean (mg/dl) Albumin, mean (g/dl)	No	213 (59.8) 6.84 3.59	88 (49.7) 3.65 3.67	125 (69.8) 10.02 3.51	*P* = 0.009 *P* = 0.085

The patients’ mean weight was 72.7 ± 14.5 kg. The mean BMI was 25.3 ± 4.2 kg/m^2,^ and %BF was 30.5 ± 6.3% (28 ± 4.9% in males and 36.4 ± 5.2% in females (*p* < 0.001)), estimated both by CUN-BAE. Patients with excess weight had lower C-reactive protein concentrations, compared with those with a BMI < 25 kg/m^2^ (3.65 vs. 10.02; *p* = 0.009, 95%CI 1.61–11.16; Table 1).

### Clinical outcomes

The clinical benefit analysis included data from 331 patients. Using RECIST criteria (v1.1.), up to 33 patients (10%) showed CR, 122 (36.9%) PR, 52 (15.7%) SD and 124 (37.5%) PD. ORR was 46.8% across all tumor types.

The median PFS was 4 months (IQR 2–8 months) and OS was 11 months (IQR 6–24 months). A total of 234 (70.7%) patients showed progression during follow-up and 204 (63.7%) patients died despite treatment with ICIs.

### Excess weight and treatment efficacy

We found no statistically significant differences in ORR between excess-weight and normal-weight patients, according to BMI (51.6% vs. 48.4%; *p* = 0.85). We analyzed the best overall response in excess-weight and normal-weight patients: 13/169 (7.7%) of the obese patients showed CR; 67/169 (39.6%) PR; 22/169 (13%) SD; and 67/169 (39.6%) PD. In contrast, 20/162 (12.3%) of the normal weight patients showed CR; 55/162 (34%) PR; 30/162 (18.5%) SD; and 57/162 (35.2%) PD. We also explored association between ORR and BMI, analyzing quartiles and quintiles, with no differences across categories or comparing extreme groups (data not shown).

The percentage of progression was 71.9% in the excess-weight group versus 67.9% in the normal-weight group (*p* = 0.439). No differences were found regarding mortality; 64.4% of patients died in the excess-weight group versus 63.1% in normal-weight patients (*p* = 0.8).

According to previous results, no differences were found between excess-%BF and normal-%BF patients in ORR (93.5% vs. 6.5%; *p* = 0.602), progression (69.7% vs. 73.7%; *p* = 0.714), or mortality (63.5% vs. 68.4%; *p* = 0.662).

### Survival outcomes

**Fig. 1:**
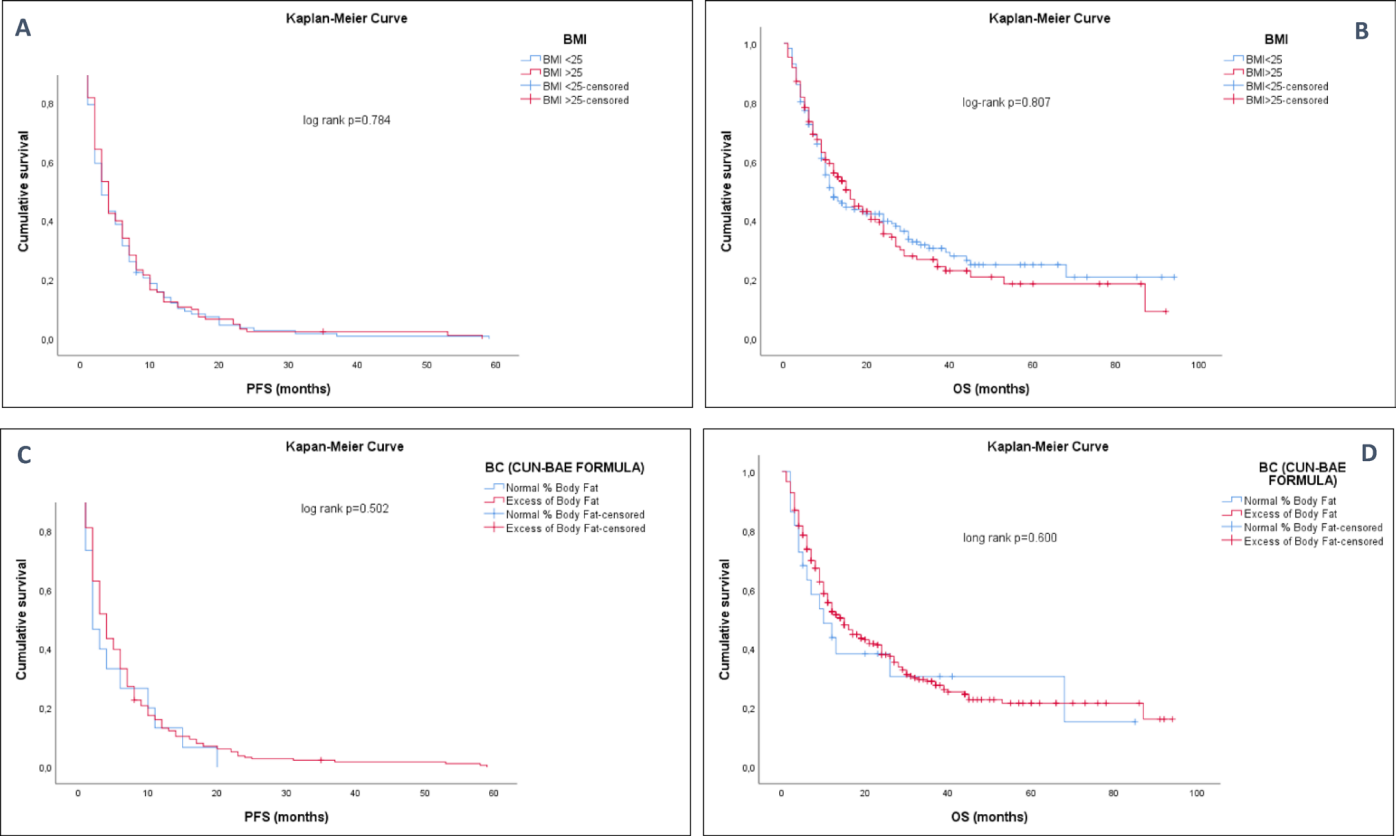
Kaplan-Meier curves in patients with advanced cancer treated with ICIs according to BMI and %BF (CUN-BAE). (**A**) Median PFS (months) according to BMI (< 25 kg/m2 vs. ≥ 25 kg/m2). (B) Median OS (months) according to BMI. (**C**) Median PFS (months) between excess-percentage of body fat group and lean patients (measured by %CUN-BAE). (**D**) Median OS (months) between excess-percentage of body fat group and lean patients (measured by %CUN-BAE)

PFS and OS were not associated with differences concerning BMI nor %BF (Fig. 1), even after adjusting by gender, age, primary tumor, smoking habit, type of ICI, ECOG, and all the clinical and demographic variables collected (Tables 2 and 3).

**Table 2:** Multivariable analyses of prognostic factors for PFS in patients with advanced cancer treated with ICIs

PFS	HR	*p* Value	95% CI	
**Gender**	0.815	0.190	0.601	1.107
**Age**	1.015	**0.027**	1.002	1.029
**Smoking habit**				
Former		0.717		
Never	0.855	0.426	0.582	1.257
Current	0.927	0.712	0.619	1.387
**ECOG**	0.565	**0.044**	0.324	0.985
**Primary tumor**				
Urothelial Cancer		0.901		
Lung cancer	0.906	0.648	0.592	1.386
Melanoma	0.929	0.787	0.546	1.581
**ICI treatment**				
Atezolizumab		0.845		
Nivolumab	0.963	0.834	0.675	1.372
Pembrolizumab	0.904	0.567	0.641	1.276
**irAEs**	1.543	**0.007**	1.126	2.114
**BMI**	0.914	0.533	0.690	1.212
**%BF (CUN-BAE)**	1.311	0.377	0.720	2.387

Gender, age, smoking habit, ECOG status, primary tumor, ICI treatment, irAEs, BMI and %BF (CUN-BAE) were evaluated in the Cox regression model

**Table 3:** Multivariable analyses of prognostic factors for OS in patients with advanced cancer treated with ICIs

OS	HR	*p* value	95% CI	
**Gender**	1.062	0.708	0.776	1.454
**Age**	1.027	**0.000**	1.013	1.041
**Smoking habit**				
Former		0.990		
Never	0.974	0.888	0.681	1.394
Current	0.994	0.977	0.679	1.457
**ECOG**	0.739	0.236	0.449	1.219
**Primary tumor**				
Urothelium		0.762		
Lung cancer	0.959	0.843	0.637	1.445
Melanoma	0.838	0.512	0.495	1.420
**ICI treatment**				
Atezolizumab		0.003		
Nivolumab	1.021	0.910	0.711	1.467
Pembrolizumab	0.607	**0.004**	0.430	0.856
**irAEs**	1.736	**0.000**	1.279	2.355
**BMI**	0.869	0.340	0.653	1.159
**%BF (CUN-BAE)**	1.498	0.186	0.823	2.729

Gender, age, smoking habit, ECOG status, primary tumor, ICI treatment, irAEs, BMI and %BF (CUN-BAE) were evaluated in the Cox regression model

It is worth noting that further PFS was shown in younger patients (HR 1.015, *p* = 0.027) with better functional status (HR 0.575, *p* = 0.044) and those who developed irAEs (HR 1.543, 95% CI 1.12–2.11; *p* = 0.007; Table 2).

Age and development of irAEs were found as predicitive factors of OS (HR 1.027, *p* < 0.001 and HR 1.736, 95% CI 1.27–2.35; *p* < 0.001, respectively; Table 3).

### Adverse event profiles

Up to 112 patients (32%) developed irAEs from the initiation of treatment, yielding a total of 128 events. Thyroid dysfunction was the most frequent irAE (*n* = 62 events, 48.4%, 57 cases grade 1, 3 cases grade 2 and 1 grade 3), followed by immune-mediated pneumonitis (*n* = 17 events, 13.2%, 1 case grade 1, 1 case grade 2, 1 case grade 4 and 14 cases grade 3). Hypothyroidism was the most common thyroid dysfunction (44/62, 71.0%; 59.1% subclinical and 40.9% overt hypothyroidism), followed by subclinical hyperthyroidism (11/62, 17.7%) and overt hyperthyroidism (7/62, 11.3%). Other irAEs were immune-mediated polyneuropathy (*n* = 9, 6 cases grade 2 and 3 cases grade 3), colitis (*n* = 7, grade 1), hepatitis (*n* = 5, 4 cases grade 3 and 1 case grade 4), arthritis (*n* = 5, 4 cases grade 1 and 1 case grade 2), interstitial nephritis (*n* = 4, grade 3), myocarditis (*n* = 3, grade 3), adrenal insufficiency (*n* = 3, grade 2), hypophysitis (*n* = 3, grade 2), cystitis (*n* = 1, grade 2), dermatitis (*n* = 1, grade 1), pancreatitis (*n* = 1, grade 4) and diabetic ketoacidosis (*n* = 1, grade 4).

### IrAEs, excess weight, and clinical outcomes

In our study, obese patients did not develop more irAEs than normal-weight patients. The lack of association was seen in the BMI and %BF analysis. Fifty-seven out of the 112 patients (50.8%) with irAEs had a BMI ≥ 25 kg/m^2^ vs. 55 patients (49.1%) with normal BMI (*p* = 0.762). Out of 238 patients without treatment-related toxicity, 117 (49.2%) were excess-weight patients and 121 (50.8%) had normal BMI (Table 1). All patients but five who developed irAEs had abnormal %BF (*p* = 0.335).

**Fig. 2:**
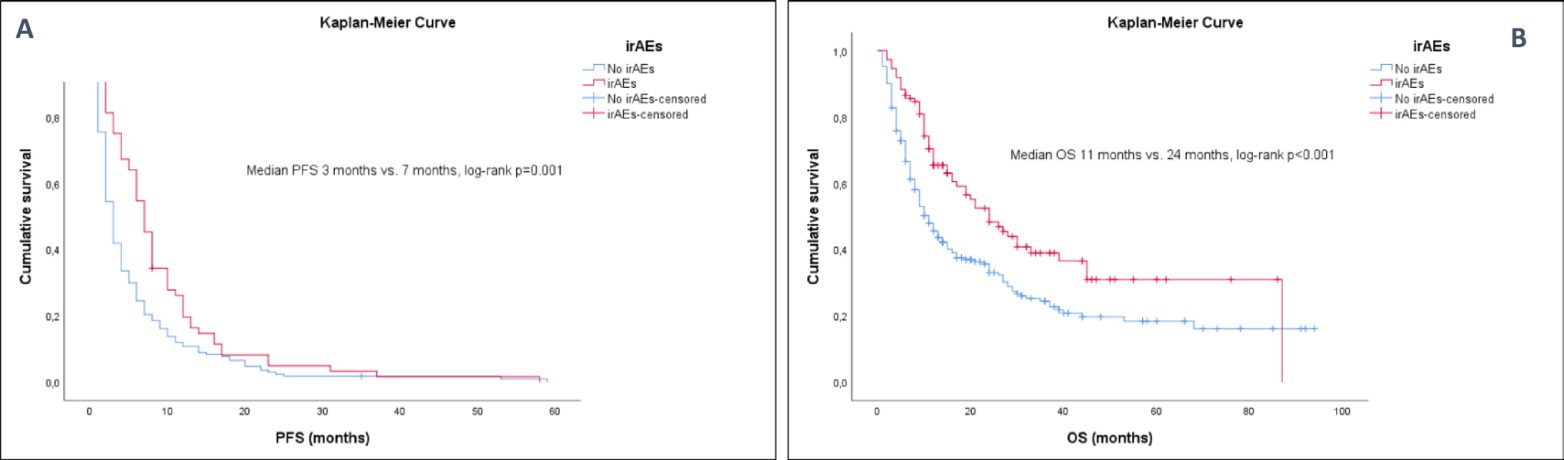
Kaplan-Meier curves in patients with advanced cancer treated with ICIs who develop irAEs versus those who do not develop irAEs. (**A**) Median of PFS (months) in patients who develop irAEs versus those who do not develop irAEs. (**B**) Median of OS (months) in patients who develop irAEs versus those who do not develop irAEs

However, the development of any-grade irAEs, regardless of BMI, %BF, age, gender, smoking habit, ECOG, type of ICI, or primary tumor, showed a better ORR, PFS, and OS than those who did not develop immunotoxicity (Fig. 2; Tables 2, 3 and 4).

**Table 4:** Relationship between objetive radiological response (ORR) and development of immune-related adverse events (irAEs) in patients with advanced cancer treated with ICIs

ORR	No irAEs (*n* = 244)	irAEs (*n* = 112)	Total	*p* value
**No ORR**	136 (77.3%)	40 (22.7%)	176	**< 0.001**
**ORR**	87 (56.1%)	68 (43.9%)	155	
**Not evaluated**	21 (84.0%)	4 (16.0%)	25	

### PD-L1 expression and obesity

PD-L1 expression was assessed in 117 out of 356 cases, all with lung cancer. Thirteen of these cases showed high PD-L1 expression (PD-L1 ≥ 50%: 11.1%), using a cutoff of 50% to define high-level expression [19, 20]. PD-L1 expression was not associated with BMI or %BF (*p* = 0.188 and *p* = 0.657, respectively), neither analyzing these variables as continuous nor as quantitative. In addition, we did not find an association between PD-L1 expression and survival outcomes (log-rank *p* = 0.808 for PFS and log-rank *p* = 0.730 for OS). Similar results were seen when using a 10% of PD-L1 expression cutoff (data not shown).

### IrAEs, excess weight, and clinical outcomes according to gender

Finally, we analyzed the possible impact of gender on the relationship between irAEs, excess weight, and clinical outcomes in patients treated with ICIs stratifying the cohort by gender. Regarding response rates, we did not find statistically significant differences between excess-weight and normal-weight men nor women using BMI (*p* = 0.471 and *p* = 0.338, respectively). However, normal-%BF was associated with higher ORR in men (81.8% vs. 47.1%, *p* = 0.024; Table 5). This association was not statistically significant in women, although the opposite tendency was observed (*p* = 0.074; Table 5). Analyzing survival rates, there were no differences in PFS or OS according to BMI/%BF in both sexes.

**Table 5:** Relationship between objetive radiological response (ORR) and estimated pecentage of body fat (%BF) in men and women.

ORR	Normal %BF	Excess %BF	Total	*p* Value
**Men**				
**No ORR**	2 (18.2%)	117 (52.9%)	119	**0.024**
**ORR**	9 (81.8%)	104 (47.1%)	113	
**Not evaluated**	2 (10.5%)	17 (89.5%)	19	
**Women**				
ORR	Normal %BF	Excess %BF	Total	*p* Value
**No ORR**	7 (87.5%)	50 (54.9%)	57	0.074
**ORR**	1 (12.5%)	41 (45.1%)	42	
**Not evaluated**	1 (16.7%)	5 (83.3%)	6	

The incidence of irAEs was not different between BMI or %BF groups in men nor women (*p* = 0.86 and *p* = 0.45 in men; *p* = 0.89 and *p* = 0.66 in women). However, the positive effect on response rates and survival of developing irAEs was also seen stratifying the cohort by gender (*p* < 0.001).

## Discussion

Given the expanding indications of ICIs in cancer treatments, there has been substantial interest in evaluating the potential clinical factors, such as gender or BMI, in predicting response to treatment with ICIs and adverse events profiles [21–31, 40–42].

In this scenario, the present single-center large study showed no better results in terms of ORR, PFS, and OS in obese patients with advanced melanoma, lung or urothelial carcinoma, even measuring excess-weight with estimated-percentage of body fat in the pooled analysis.

On the other hand, the incidence of irAEs in this cohort was independent of weight, as we have shown in multivariate analysis adjusting for primary tumor type, ICI regimen, age, sex, BMI, and BF.

Our findings further suggest that the relation between obesity and outcomes in patients with advanced cancers treated with ICIs might vary by gender, showing a improved significant response rate in lean men versus excess-weight men and the opposite association in women, although this one was not statistically significant.

Recent studies have drawn inconsistent conclusions suggesting obesity as a modifiable factor in this setting [26, 27, 31]. Although obesity is associated with an increased risk of cancer development, mainly as a result of chronic inflammation, which in turn induces T cell dysfunction, the related T cell polarization toward a pre-exhaustation phenotype and the upregulation of PD-1 are associated with improved response rates to anti-PD(L)1 therapy. Some retrospective observational studies have found a positive association between high BMI ​​and greater OS and PFS in patients with different primary tumors (melanoma, lung or kidney carcinomas) treated with ICIs [19–23, 40]. McQuade et al. [24] associated BMI with better PFS and OS in male patients with melanoma treated with immunotherapy. In line with this observation, a recent study by Rogado J et al. [25] also showed that patients with excess weight and advanced cancer who receive single-agent anti-PD-1 antibody therapy exhibit a significantly improved clinical outcome compared with patients with normal weight. This association was especially marked when BMI and irAEs were considered in combination. The latest publication that support previous results were published by Alden et al. [44], showing this association in relation to different immune response to ICIs in obese and non-obese patients.

Despite the previous results that indicate a favorable efficacy with ICIs in patients with higher BMI, contradictory findings have also been reported [26, 27]. A couple of observational studies identified no differences in treatment outcomes according to BMI, including a large cohort of patients with different types of advanced primary tumors [31] or a sample that exhibited a lower survival rate when they had a high BMI [32]. The discrepancy in the results could be due to a variety of factors that modulate the response to ICIs. These could include heterogeneity in cancer type, age, and BMI threshold. However, it is hard to define the predictive value of BMI for outcomes after ICI therapy.

In fact, we know that, although BMI is widely used to define obesity, it is not always a good indicator of obesity since a high percentage of patients categorized by BMI as being in the overweight range could be categorized as obese when assessed more accurately by body composition (BC) [16]. Therefore, it is advisable to study other parameters related to obesity, such as BC. As the present study was retrospectively conducted, we used CUN-BAE to estimate the body fat percentage in this population. As previously reported [15], this is an easy-to-apply and validated predictive equation that may be used as an initial screening tool in clinical practice. Again, our analysis did not find a significant statistical association between BMI/%BF and clinical outcomes in any of the three malignancies, however, analyzing data stratified by gender, it was revealed a positive association between normal percentage of estimated body fat and improved reponse to ICIs limited to male patients, suggesting a negative impact of excess body fat in clinical outcomes for men.

Differences in response according to gender were shown before by Mcquade et al. [24] The differences between obesity and response to ICIs by sex reported here also suggest a potential hormonal mediator of the adiposity effects. Sex and gender (biological and behavioral differences associated with being male or female) affect the development and function of the immune system and in recent years, differences in the molecular mechanisms of the anticancer immune response between females and males with different types of tumors have been recognized [33]. The hormonal environment can significantly influence the regulation of B cell activity, antibody production, and vaccine efficacy. Estrogens also have been shown to modulate the PD-1/PD-L1 pathway in T cells, contributing to greater autoimmune responses in females as compared with males [34]. It remains to be determined whether the effects of sex steroids, including testosterone, on regulatory pathways in T cells account for differences in the efficacy of checkpoint therapies between males and females, and whether such sex-related differences vary across the life course. Nonetheless, a recent meta-analysis found no significant sex differences in the response to immune checkpoint inhibitors when OS and PFS were used as endpoints [35].

To the best of our knowledge, this is one of the largest retrospective studies using estimated BC to analyze the effect of excess weight on ICI outcomes. In this context, several studies have considered BC to examine this relationship [29, 33, 36–40]. Most have concluded that fat tissue content is related to chronic inflammation parameters and focus their results on the prognostic negative impact of sarcopenia, showing reduced survival rates [33, 36, 38]. In the study published by Xiao et al. [36], BMI was not associated with survival outcomes. In keeping with our findings, Young AC et al. [41], in the largest study examining the association between BC and outcomes in patients with metastatic melanoma receiving ICI, showed no association with outcomes and BMI but identified trends towards worse outcomes in patients with higher adiposity parameters and lower muscle quality and quantity. In our study BMI and %BF was analysed at a single timepoint (therapy initiation) so we cannot rule out potential antecedent weight loss due to illness, and future studies should include longitudinal BMI and %BF assessment. However, underweight was rare in out cohort and such patients were excluded from the analysis. Moreover, ECOG, C reactive protein and albumin concentrations were similar in both groups by BMI/%BF categories, so it is expected that patients with normal BMI and normal %BF were not cachectic at baseline.

On the other hand, a potential role of BMI in irAE development has also been suggested [21, 22, 41–43]. Nevertheless, our data suggest no increased risk of irAEs in obese patients treated with ICIs, as other studies have shown [22, 31]. The largest study to date on the impact of BMI on irAEs, involving 1434 patients in a pooled analysis of phase II/III trials of atezolizumab in NSCLC, was by Kichendasse et al. [22] and, similarly, their results showed no association.

These inconsistencies have several possible explanations. First of all, BMI is not a reliable marker of obesity in most cases. Also, the role of adipose tissue in cancer progression and response to ICI may be dynamic, with tumors in obese individuals being initially more vulnerable to ICI but ultimately associated with irreversible T-cell exhaustion and worse outcomes [44]. This paradoxical phenomenon, known as the ‘obesity paradox,’ has been previously reported in cancer patients, wherein obesity is associated with an increased risk of cancer development but, in certain contexts -particularly in the absence of sarcopenia- may be linked to better outcomes [45].

There were several limitations in this study. First, this was a retrospective study; therefore, subsequent prospective studies must be conducted to verify these results. Second, we could only estimate BC because it was retrospective so other direct methods were not utilized. Although the CUN-BAE formula has been validated as an accurate tool for estimating body fat [15], it does not constitute a direct measurement method. Exploring other anthropometric indices such as waist circumference (WC) or direct BC methods of visceral adiposity by CT may provide valuable confirmation of these results. Third, our study participants were heterogeneous and most had excess-BF, which may have introduced a bias. Also, a smaller number of women were included in the cohort, so it could have limited the statistical power to detect associations in this group. Finally, we lost a number of patients during follow-up because they did not undergo CT or irAEs ocurred when they were followed-up in a different center.

On the other hand, our sample size was significant, we included a large series of patients with three different types of solid tumors and receiving three different ICI regimens. We found no differences, according to BMI, even adjusting by gender, tumor type, drug and other clinical and analytical variables that were collected (such as inflammatory or nutritional markers: CRP, L/N ratio, glucose, lipid profile, serum albumin levels, etc.). However, the association between %BF and clinical response, evaluated by ORR, was only seen in men, supporting the hypothesis of the hormonal influence in the immune antitumoral response.

## Conclusions

Our analysis suggests that obesity measured by BMI is not associated with treatment response or survival outcomes in patients with advanced cancers treated with ICIs. However, lean men showed higher ORR compared to men with excess body fat. Moreover, the development of irAEs may serve as a prognostic response factor.

These findings support the need to consider body composition, gender and irAEs as stratification factors in trials, and to investigate the biological mechanisms underlying this association.

## Data Availability

The data that support the findings of this study are available from the corresponding author, [SO], upon reasonable request.
